# Analysis of the Phenotype of *Mycobacterium tuberculosis*-Specific CD4+ T Cells to Discriminate Latent from Active Tuberculosis in HIV-Uninfected and HIV-Infected Individuals

**DOI:** 10.3389/fimmu.2017.00968

**Published:** 2017-08-10

**Authors:** Catherine Riou, Natacha Berkowitz, Rene Goliath, Wendy A. Burgers, Robert J. Wilkinson

**Affiliations:** ^1^Division of Medical Virology, Faculty of Health Sciences, Department of Pathology, University of Cape Town, Cape Town, South Africa; ^2^Institute of Infectious Disease and Molecular Medicine, University of Cape Town, Cape Town, South Africa; ^3^Wellcome Center for Infectious Diseases Research in Africa, Institute of Infectious Disease and Molecular Medicine, University of Cape Town, Cape Town, South Africa; ^4^Department of Medicine, Imperial College London, London, United Kingdom; ^5^Francis Crick Institute, London, United Kingdom

**Keywords:** *Mycobacterium tuberculosis*, HIV, latent tuberculosis infection, active tuberculosis, *Mycobacterium tuberculosis*-specific CD4 response

## Abstract

Several immune-based assays have been suggested to differentiate latent from active tuberculosis (TB). However, their relative performance as well as their efficacy in HIV-infected persons, a highly at-risk population, remains unclear. In a study of 81 individuals, divided into four groups based on their HIV-1 status and TB disease activity, we compared the differentiation (CD27 and KLRG1), activation (HLA-DR), homing potential (CCR4, CCR6, CXCR3, and CD161) and functional profiles (IFNγ, IL-2, and TNFα) of *Mycobacterium tuberculosis* (Mtb)-specific CD4+ T cells using flow cytometry. Active TB disease induced major changes within the Mtb-responding CD4+ T cell population, promoting memory maturation, elevated activation and increased inflammatory potential when compared to individuals with latent TB infection. Moreover, the functional profile of Mtb-specific CD4+ T cells appeared to be inherently related to their degree of differentiation. While these specific cell features were all capable of discriminating latent from active TB, irrespective of HIV status, HLA-DR expression showed the best performance for TB diagnosis [area-under-the-curve (AUC) = 0.92, 95% CI: 0.82–1.01, specificity: 82%, sensitivity: 84% for HIV− and AUC = 0.99, 95% CI: 0.98–1.01, specificity: 94%, sensitivity: 93% for HIV+]. In conclusion, these data support the idea that analysis of T cell phenotype can be diagnostically useful in TB.

## Introduction

With almost 2 million deaths and over 10 million new cases in 2015, tuberculosis (TB) remains a global health priority. In southern Africa, the HIV pandemic fuels the TB burden, where over 50% of new TB cases are HIV associated (1). Control of the TB epidemic has been impaired by the lack of an effective vaccine but also the scarcity of sensitive and rapid diagnostic tests that would facilitate early treatment initiation and reduce transmission, morbidity, and mortality (2). To date, the gold standard for TB diagnosis relies on detection of *Mycobacterium tuberculosis* (Mtb) using microscopy, culture, or molecular methods (3, 4). While being of relatively high specificity, all these techniques require either sputum or a specimen from the site of disease. This makes diagnosis of pulmonary TB in patients with negative sputum smears or a non-productive cough, or extrapulmonary TB, difficult. This limitation is especially relevant in HIV-infected persons, where immunosuppression associates with reduced cavitation, limiting the sensitivity of sputum-based assays (5). Thus, alternate host response-based diagnostics are much needed to distinguish latent tuberculosis infection (LTBI) from active TB (aTB), particularly for those most at risk, such as HIV-1-infected individuals.

Several attributes of Mtb-specific CD4+ T cells have been shown to efficiently delineate LTBI and aTB in HIV-uninfected individuals. This includes their polyfunctional capacities (6–11), memory differentiation profile (12–16), and activation status (17). However, the relative performance of these markers has not yet been compared and their relevance in HIV-infected individuals remains to be determined. We recently showed that the activation profile of Mtb-specific CD4+ T cells, unlike their functional profile, reflects disease activity irrespective of HIV status (18).

In this study, we defined the phenotypic and functional characteristics of peripheral Mtb-specific CD4+ T cells in individuals with distinct HIV status and TB disease activity. Our aims were to (i) gain a better understanding of the detrimental impact of HIV infection on Mtb adaptive immunity, (ii) validate and compare the performance of previously described potential biomarkers to discriminate LTBI from aTB in HIV-uninfected persons, (iii) identify new potential markers, and finally (iv) define the performance of these markers in HIV-infected individuals.

## Materials and Methods

### Study Participants

Study participants (*n* = 81) were recruited from the Ubuntu Clinic, Khayelitsha (Cape Town, South Africa). Participants were divided into four groups based on their HIV-1 and TB status: HIV−/LTBI (*n* = 20), HIV+/LTBI (*n* = 21), HIV−/aTB (*n* = 20), and HIV+/aTB (*n* = 20). LTBI was diagnosed based on a positive IFNγ release assay (QuantiFERON^®^-TB Gold In-Tube), no symptoms of aTB and a negative Mtb-sputum (GeneXpert). Diagnosis of aTB was based on clinical symptoms and positive Mtb-sputum. All aTB cases were fully drug sensitive and TB treatment-naïve. All HIV-infected individuals were antiretroviral treatment (ART)-naive. The study was approved by the University of Cape Town Human Research Ethics Committee (No. 896/2014).

### Cell Preparation and Activation

PBMC were isolated by Ficoll-Hypaque density gradient centrifugation (GE Health care), cryopreserved and stored until needed. Cryopreserved PBMC were thawed and rested in RPMI 1640 containing 10% heat-inactivated FCS prior to antigen stimulation. PBMC were stimulated using Mtb cell lysate (strain H37Rv, BEI resources) or early secretory antigenic target (ESAT)-6/culture filtrate protein (CFP)-10 peptide pool consisting of 17 and 16 peptides covering the entire 6-kDa ESAT-6 and 10-kDa CFP-10, respectively (2 µg/ml, Peptide Synthetics) or a pool of cytomegalovirus virus (CMV) peptides consisting of 138 peptides covering the entire pp65 protein (2 µg/ml). Brefeldin A (10 µg/ml, Sigma) was added 3 h after Mtb lysate stimulation or at the time of peptides addition. All stimulations were performed in the presence of co-stimulatory antibodies, anti-CD28 and anti-CD49d (both at 1 µg/ml; BD) for 16 h.

### Cell Staining

After stimulation, cells were washed, stained with LIVE/DEAD^®^ Fixable Near-IR Stain (Invitrogen) and, subsequently, surface stained with the following antibodies: CD14-APC/Alexa Fluor 750 (Invitrogen) and CD19-APC/Alexa Fluor 750 (Invitrogen), CD4-FITC (BD), CD27-BV711 (BD), CCR4-BV510 (Biolegend), CCR6-BV605 (Biolegend), CXCR3-PE-Cy7 (BD Biosciences), KLRG1-PerCP/eFluor 710 (eBioscience), and HLA-DR-PE (BD). Cells were then fixed and permeabilized using Cytofix/Cytoperm buffer (BD) and stained with CD3-BV650 (BD), IL-2-PE/Dazzle™ (Biolegend), TNFα-eFluor 450 (eBioscience), and IFNγ-Alexa Fluor 700 (BD). Finally, cells were washed and fixed in 1% formaldehyde in PBS. Samples were acquired on a LSR-II (BD) and analyses were performed using FlowJo (Treestar). A positive IFNγ response was defined as at least twice the background measured in the presence of co-stimulatory antibodies without antigen. Cell polyfunctionality was analyzed using Pestle and Spice software (19). The gating strategy is presented in Figure S1 in Supplementary Material.

### Statistical Analysis

Statistical analyses were performed using GraphPad Prism. The Mann–Whitney *U* test and Wilcoxon-matched pairs test were used for unmatched and paired samples, respectively, and the Kruskal–Wallis ANOVA using Dunn’s test for multiple comparisons. Correlations were performed using the Spearman rank test. Logistic regression followed by receiver operating characteristic (ROC) curve analysis, which plots sensitivity (true-positive rates) vs 1-specificity (false-positive rates), was used to evaluate the performances of each marker to identify aTB and to establish probability cutoff values.

## Results

### Magnitude of Mtb-Specific CD4+ T Cells in the Context of LTBI and aTB in HIV-Uninfected and HIV-Infected Individuals

**Figure 1:**
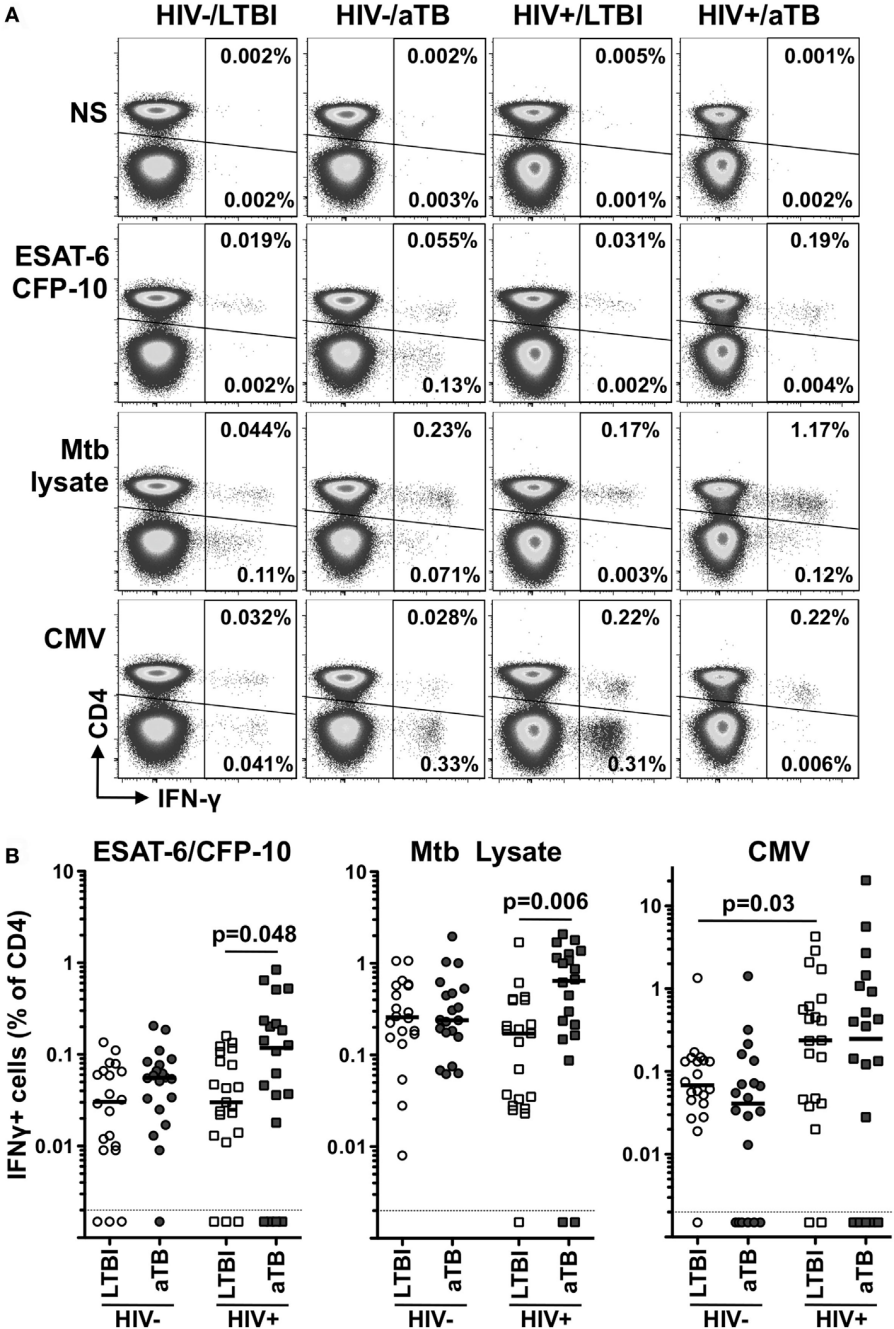
Comparison of the frequencies of early secretory antigenic target (ESAT)-6/culture filtrate protein (CFP)-10-, *Mycobacterium tuberculosis* (Mtb) lysate-, or cytomegalovirus virus (CMV)-specific IFNγ+ CD4+ T cells in individuals with distinct HIV and tuberculosis (TB) disease status. **(A)** Representative flow cytometry plots of the expression of IFNγ from CD4+ and CD3+ CD4− T cells after stimulation with ESAT-6/CFP-10, Mtb lysate and CMV antigens in one individual from each clinical group. NS corresponds to unstimulated PBMC. The frequency of IFNγ-producing cells expressed as a percentage of the total CD3+ CD4+ or total CD3+ CD4− T cell population is indicated. **(B)** Frequency of IFNγ-producing CD4+ T cell in response to ESAT-6/CFP-10, Mtb lysate or CMV in HIV−/latent tuberculosis infection (LTBI) (*n* = 20), HIV−/active TB (aTB) (*n* = 20), HIV+/LTBI (*n* = 21), and HIV+/aTB (*n* = 20). Bar represents the median. Symbols shown below the horizontal dashed line correspond to non-responders. Statistical comparisons were performed using a Mann–Whitney *t*-test.

To better understand the profile of Mtb-specific T cell responses, we first defined the frequency of Mtb-specific CD4+ T cells in 81 participants grouped according to HIV-1 and TB status (Table 1). We measured IFNγ production in response to ESAT-6/CFP-10 peptide pool and Mtb cell lysate. In parallel, we also monitored T cell responses to CMV, used as an unrelated pathogen for comparison (Figure 1A).

**Table 1:** Clinical characteristics of the four study groups.

	HIV−/LTBI	HIV+/LTBI	HIV−/aTB	HIV+/aTB
*n*	20	21	20	20
CD4 count (cells/mm^3^)^a^	853 [694–1,054]	563 [440–722]	604 [515–838]	149 [55–212]
Log_10_ HIV viral load (copies/ml)^a^	na	4.00 [3.14–4.92]	na	5.35 [4.26–5.95]
Age	26 [20–31]	31 [27–43]	34 [27–42]	38 [29–46]
Gender (F/M)	10/10	18/3	1/19	9/11
ESAT-6/CFP-10 responders (%)^b^	17/20 (85%)	18/21 (86%)	19/20 (95%)	16/20 (80%)
Mtb lysate responders (%)^b^	20/20 (100%)	20/21 (95%)	20/20 (100%)	18/20 (90%)
CMV responders (%)^b^	19/20 (95%)	19/21 (90%)	14/20 (70%)	14/20 (70%)

*^a^Median and interquartile ranges*.

*^b^Number of individuals with a positive IFNγ+ CD4+ T cell response to the specified antigen*.

*F, female; M, male, LTBI, latent tuberculosis infection; aTB, active tuberculosis disease; ESAT, early secretory antigenic target; CFP, culture filtrate protein; Mtb, Mycobacterium tuberculosis; CMV, cytomegalovirus virus*.

For all tested pathogens, the frequency of IFNγ responding CD4+ T cells was comparable in HIV-uninfected individuals, irrespective of their TB status (Figure 1B).

Conversely, in HIV-infected individuals, Mtb lysate and ESAT-6/CFP-10 responses were significantly elevated in participants with aTB disease when compared to LTBI (median: 0.64 vs 0.24% for Mtb lysate and 0.13 vs 0.03% for ESAT-6/CFP-10, respectively). Such differences were not observed for CMV-specific responses (Figure 1B). This surprising elevation of the frequency of Mtb-specific CD4+ T cells in HIV+/aTB individuals, despite the severe lymphopenia characterizing this group (median CD4 count: 149 cells/mm^3^), suggests that active Mtb replication promotes Mtb-specific CD4+ T cell expansion in ART naïve HIV+/aTB patients. Moreover, the elevated frequency of ESAT-6/CFP-10-specific CD4+ T cells observed in the HIV+/LTBI group could appears to differ from a previous study showing a rapid depletion of Mtb-specific CD4+ T cells in subjects with LTBI within 1 year after HIV infection (20). However, in our study cohort, recruited from a highly TB endemic area, recurrent Mtb exposure, and the relatively well-preserved CD4+ T cell count in HIV-infected individuals with LTBI (median: 563 cells/mm^3^) could account for the conservation of Mtb-specific CD4+ T cells.

In addition, we also detected IFNγ production in response to ESAT-6/CFP-10 stimulation originating in the CD3+ CD4− compartment. Such responses were predominantly observed in aTB patients [75% (15/20) for the HIV− group and 60% (12/20) for the HIV+ group]. On the contrary, only one-fifth of latently infected persons exhibited detectable ESAT/CFP-specific IFNγ+ CD4− CD3+ T cell responses [20% (4/20) for HIV− and 19% (4/21) for HIV+], as previously reported (21) (data not shown).

### Alteration of the Phenotype of Mtb-Specific CD4+ T Cells during Active TB Infection

**Figure 2:**
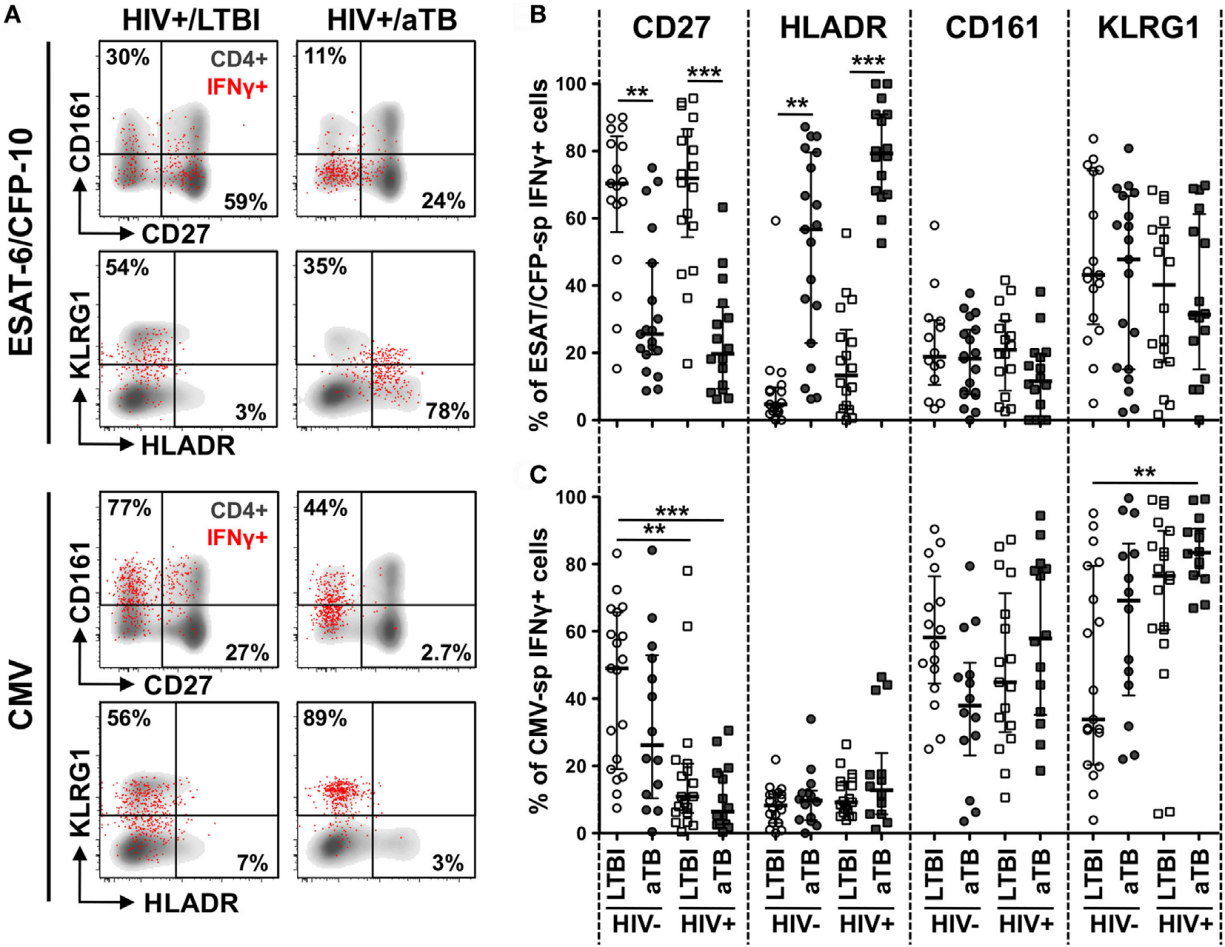
Comparison of the phenotype of early secretory antigenic target (ESAT)-6/culture filtrate protein (CFP)-10- and cytomegalovirus virus (CMV)-specific IFNγ+ CD4+ cells in individuals with HIV and/or active TB (aTB). **(A)** Representative examples of memory, homing, and activation profiles of IFNγ+ CD4+ T cells (red) and total CD4+ T cells (gray) in one latent tuberculosis infection (LTBI)/HIV−, one LTBI/HIV+, and one aTB/HIV+ individual. **(B,C)** Expression of CD27, HLA-DR, CD161, and KLRG1 on IFNγ+ CD4+ T cells in response to ESAT-6/CFP-10 **(B)** and CMV **(C)** from LTBI/HIV− (open circle), aTB/HIV− (closed circle), LTBI/HIV+ (open square), and aTB/HIV+ (closed square) individuals. Bars represent the median and interquartile range. Statistical comparisons were performed using a one-way ANOVA Kruskal–Wallis test. ** < 0.01, *** < 0.001.

We next compared the phenotype of Mtb- and CMV-responding IFNγ+ CD4+ T cells during latent and aTB disease. We focused on the memory differentiation profile (CD27), the activation status (HLA-DR and KLRG1) and the homing potential (CCR4, CCR6, CD161, and CXCR3) of these cells, as these parameters are likely to be important features regulating T cell function (Figure 2A).

In the context of aTB disease (irrespective of HIV infection), ESAT-6/CFP-10-specific IFNγ+ CD4+ T cells were characterized by significantly lower expression of CD27 (median: 26% for HIV− and 20% for HIV+) when compared to persons with LTBI (70% for HIV− and 72% for HIV+). Concomitantly, HLA-DR expression on these cells was significantly elevated in persons with aTB (median: 57% for HIV− and 79% for HIV+) compared to LTBI (5% for HIV− and 13% for HIV+) (Figure 2B). No differences in the expression of KLRG1 or any of the chemokine receptors tested were observed among Mtb-specific CD4+ T cells in the four clinical groups (Figure 2B; Figure S3 in Supplementary Material). HIV infection *per se* did not significantly alter the phenotype of ESAT-6/CFP-10-specific IFNγ+ CD4+ T cells. However, in LTBI, there was a trend toward increased HLA-DR expression on these cells in the HIV-infected persons compared HIV-uninfected participants. Notably, in the LTBI/HIV+ group, HLA-DR expression on Mtb-specific IFNγ+ CD4+ T cells mirrors HLA-DR expression in the whole CD4 compartment (*p* = 0.03, *r* = 0.49, data not shown), suggesting that during LTBI, elevated activation of Mtb-specific CD4+ T cells in probably driven by HIV-induced systemic immune activation. Similar results were obtained for Mtb lysate-specific IFNγ+ CD4+ T cells (Figure S2A in Supplementary Material).

Changes observed during aTB were restricted to CD4+ T cells targeting Mtb antigens, as the phenotypic profile of CMV-specific IFNγ+ CD4+ T cells was comparable between the LTBI and aTB groups (Figure 2C; Figure S3 in Supplementary Material). However, unlike the Mtb-specific response, HIV altered CMV-specific CD4+ T cell profile differently, inducing memory cell maturation. Indeed, CD27 expression was reduced on CMV-specific IFNγ+ CD4+ T cells in HIV-infected persons (median: 11% in LTBI and 6% in aTB) when compared to HIV-uninfected (49% in LTBI and 26% in aTB) (Figure 2C).

Overall, these results show that aTB disease associates with the activation and memory differentiation of Mtb-specific CD4+ T cells; these cellular features would be consistent with ongoing bacterial replication.

### Functional profile of Mtb-Specific CD4+ T Cells in the Context of LTBI and aTB Infection in HIV-Uninfected and HIV-Infected Individuals

**Figure 3:**
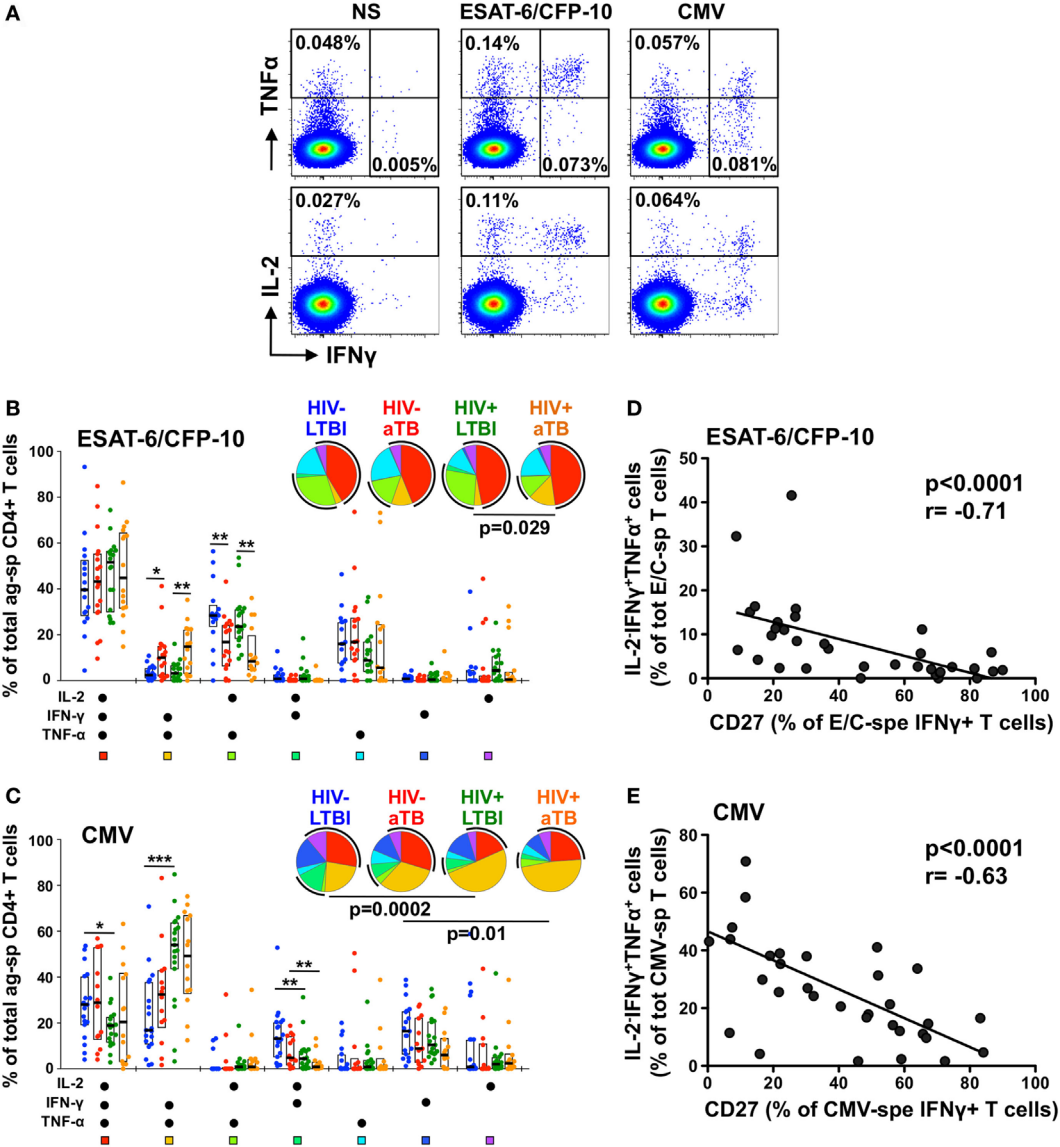
Polyfunctional capacity of early secretory antigenic target (ESAT)-6/culture filtrate protein (CFP)-10- and cytomegalovirus virus (CMV)-specific CD4+ T cells in individuals with distinct HIV and tuberculosis (TB) disease status. **(A)** Representative dot plots of the expression of TNFα, IFNγ, and IL-2 from CD4+ T cells after stimulation with ESAT-6/CFP-10 or CMV peptide pool in one latent tuberculosis infection (LTBI)/HIV− participant. NS corresponds to unstimulated PBMC. The frequency of cytokine-producing cells expressed as a percentage of the total CD4+ T cell population is indicated. **(B,C)** Graphs and pie charts representing the polyfunctional profile of CD4 T cells in response to ESAT-6/CFP-10 **(B)** and CMV **(C)**. Each section of the pie chart represents a specific combination of cytokines, as indicated by the color at the bottom of the graph. The black arc on the pies corresponds to IL-2-producing cells. Horizontal bars depict the median with interquartile ranges indicated. Statistical comparisons were performed using a Wilcoxon rank-sum test. *p < 0.05, **p < 0.01, ***p < 0.001. **(D,E)** Relationship between the CD27 expression on antigen-specific IFNγ+ CD4+ T cells and the functional capacity of antigen-responding CD4+ T cells in HIV-uninfected individuals with latent and active TB (aTB). Correlations were tested by a two-tailed non-parametric Spearman rank test.

To define whether the phenotypic changes observed in the context of aTB disease (or HIV infection) affect cell functions, we next compared the polyfunctional potential of CD4+ T cells in response to ESAT-6/CFP-10 and CMV antigens in the four clinical groups. We measured the expression of IL-2, IFNγ, and TNFα (Figure 3A).

While the majority of ESAT-6/CFP-10-responding cells were polyfunctional (co-expressing IL-2, IFNγ, and TNFα) in all study groups, Mtb-specific CD4+ T cells in persons with aTB disease (irrespective of HIV status) were characterized by a significant elevated proportion of IL-2−IFNγ+TNFα+ cells, with a concomitant decrease in the proportion of IL-2+IFNγ−TNFα+ cells (Figure 3B). Similar functional profiles were obtained in response to Mtb lysate (Figure S2B in Supplementary Material). For CMV-specific CD4+ T cells, no functional differences were observed between the HIV−/LTBI and HIV−/aTB groups. HIV infection, however, significantly skewed CMV-specific CD4+ response profiles, inducing the expansion of IL-2−IFNγ+TNFα+ cells with a concomitant contraction of IL-2+IFNγ+TNFα+ and/or IL-2+IFNγ+TNFα− cells (Figure 3C).

We then assessed whether these functional differences were related to specific cell phenotypic characteristics. Figures 3D,E show that in HIV-uninfected individuals, CD27 expression on antigen-specific IFNγ+ CD4+ T cells correlated negatively with the proportion of IL-2−IFNγ+TNFα+ cells for both Mtb and CMV responses (*p* < 0.0001). Similar associations were observed for HIV-infected persons (data not shown) and for Mtb lysate-responding CD4+ T cells (Figure S2C in Supplementary Material). This indicates that the differentiation of antigen-specific CD4+ T cells into effector memory or effector cells (i.e., cells lacking CD27 expression), as a result of Mtb replication for Mtb-specific cells or HIV infection for CMV-specific CD4+ T cells, partially attenuated their capacity to secrete IL-2.

**Figure 4:**
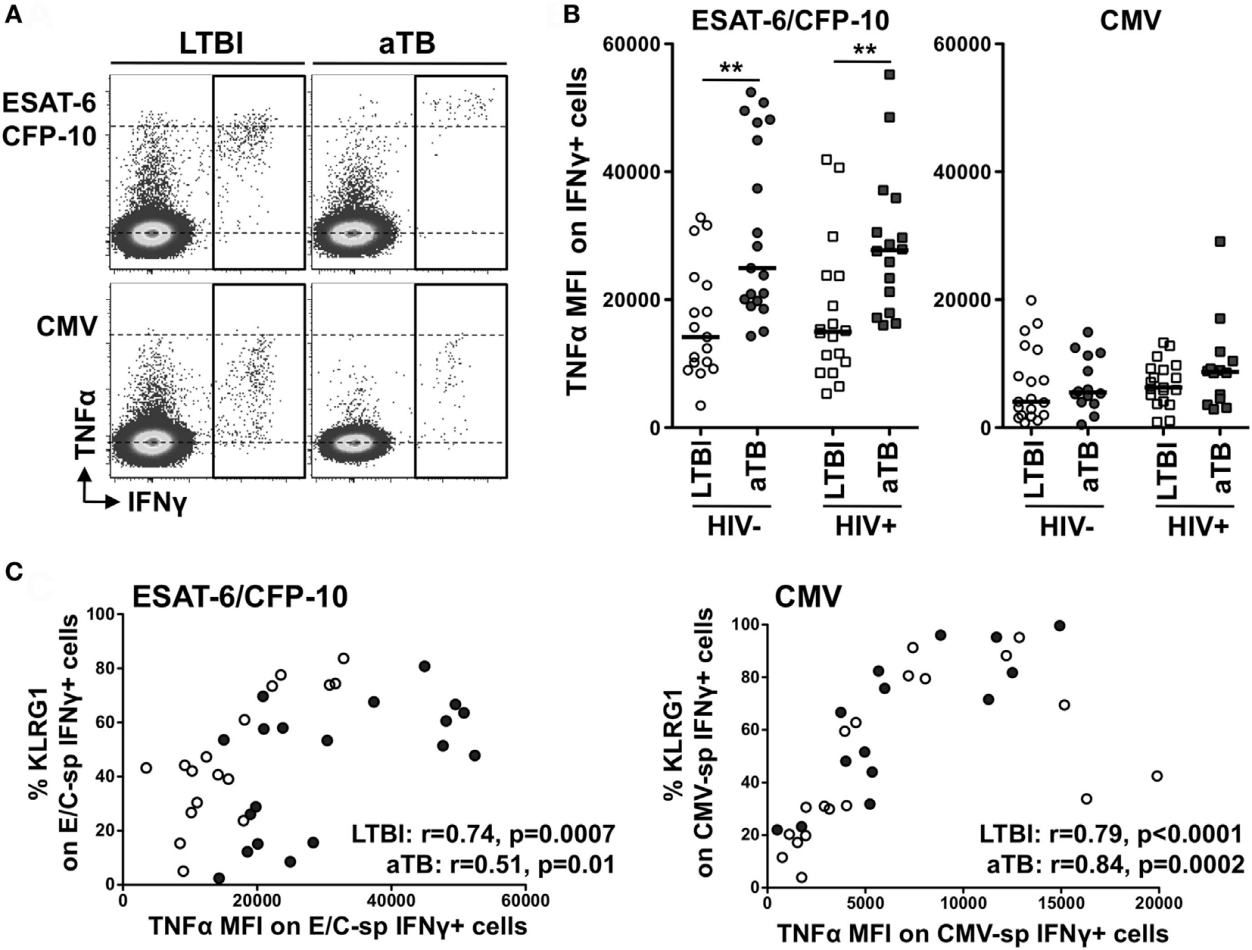
Comparison of TNFα expression levels within IFNγ+ CD4+ T cells in response to *Mycobacterium tuberculosis* and cytomegalovirus virus (CMV) antigens between the four clinical groups. **(A)** Representative example of TNFα production in response to early secretory antigenic target (ESAT)-6/culture filtrate protein (CFP)-10 or CMV in one HIV-uninfected individual with latent tuberculosis infection (LTBI) and one HIV-uninfected individual with active TB (aTB). **(B)** TNFα expression levels [expressed as median fluorescence intensity (MFI)] in antigen-specific IFNγ+ CD4+ T cells in the four clinical groups. Statistical comparisons were performed using a Mann–Whitney *t*-test between LTBI and aTB groups. ** *p* < 0.01. **(C)** Relationship between TNFα MFI in antigen-specific IFNγ+ CD4+ T cells and the expression of CD27 or KLRG1 on these cells. Correlations were tested by a two-tailed non-parametric Spearman rank test.

Finally, further analysis of the functional profile of Mtb-specific cells revealed that the median fluorescence intensity (MFI) of TNFα in ESAT-6/CFP-10-specific IFNγ+ CD4+ T cells was significantly higher in aTB (regardless of HIV infection), when compared to LTBI (median MFI: 24,920 vs 14,180 in HIV− and 27,720 vs 14,980 in HIV+, respectively) (Figures 4A,B). A similar pattern of TNFα expression level was also observed in response to Mtb lysate between the LTBI and aTB groups (Figure S2D in Supplementary Material), but no such differences were detected for CMV-specific cells (Figure 4B). Interestingly, MFI levels of TNFα in Mtb- and CMV-specific IFNγ+ CD4+ T cells in HIV-uninfected individuals were positively associated with the expression of KLRG1 on these cells (Figure 4C). Of note, similar results were obtained when these analyses were restricted to polyfunctional cells (i.e., cells co-expressing IL-2, IFNγ, and TNFα) (Figure S4 in Supplementary Material). This shows that the elevated TNFα MFI within Mtb-specific CD4+ T cells in individuals with aTB was not simply related to an overall increase in the proportion of cell producing TNFα, but rather reflects an intrinsic increase in the pro-inflammatory potential of these cells.

These data suggest that specific functional changes occur in Mtb-specific cells in aTB compared to LTBI, and these can be linked to phenotypic changes. Moreover, during aTB disease, Mtb-specific responses become highly pro-inflammatory and such characteristics appear to be inherently linked to KLRG1 expression on these cells.

### Markers Discriminating Latent from aTB

**Figure 5:**
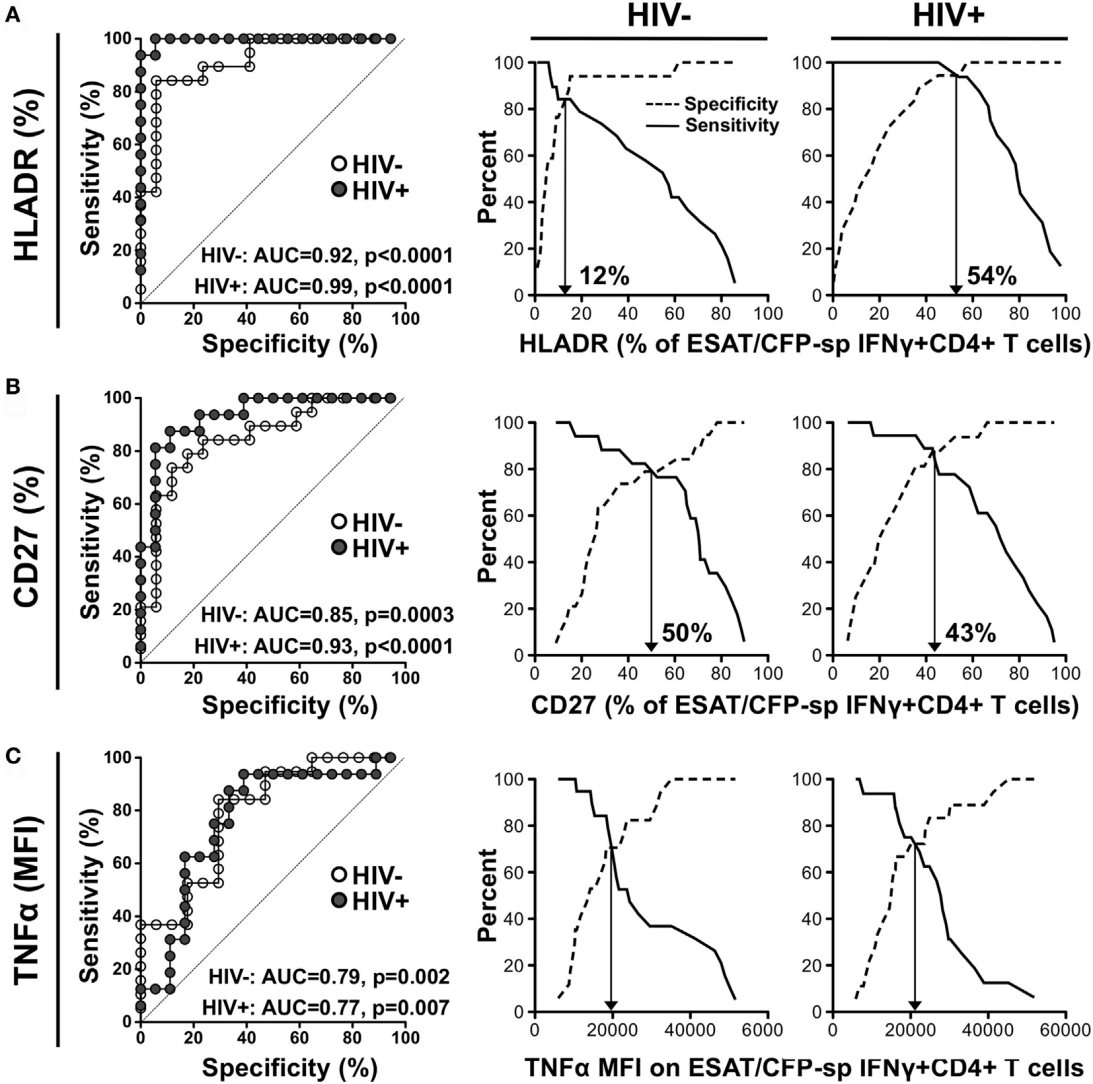
Comparison of the performances of distinct characteristics of the *Mycobacterium tuberculosis*-specific CD4+ T cell response to discriminate latent from active tuberculosis. Receiver operating characteristics (ROCs) curves and corresponding specificity/sensitivity cross-over plots for HLA-DR **(A)**, CD27 **(B)**, and TNFα **(C)** expression in early secretory antigenic target (ESAT)-6/culture filtrate protein (CFP)-10-specific IFNγ+ CD4+ T cells to discriminate between latent tuberculosis infection (LTBI) and active TB (aTB) in HIV-uninfected (open dots) and HIV-infected (closed dots) individuals. HLA-DR and CD27 are expressed as % of ESAT-6/CFP-10-specific IFNγ+ CD4+ T cells and TNFα expression is expressed as corrected median fluorescence intensity (MFI) [i.e., (TNFα MFI on ESAT-6/CFP-10-specific IFNγ+ CD4+ T cells) − (TNFα MFI on total CD4+ cells)]. For the ROC curves, the area-under-the-curve (AUC) and *p*-value are shown. The dotted line depicts an AUC of 0.5, representing a random test. For the cross-over plots, the vertical arrow represents the optimal threshold to distinguish between LTBI and aTB.

We next assessed which parameter best distinguished latent from aTB by generating ROC curves from the data obtained in ESAT-6/CFP-10 responders. HLA-DR expression on ESAT-6/CFP-10-specific IFNγ+ CD4+ T cells showed the best capability to discriminate latent from aTB, irrespective of HIV status [*p* < 0.0001, area-under-the-curve (AUC) = 0.92, 95% CI: 0.82–1.01, specificity: 82%, sensitivity: 84% for HIV− and *p* < 0.0001, AUC = 0.99, 95% CI: 0.98–1.01, specificity: 94%, sensitivity: 93% for HIV+]. However, the optimum cutoff values discriminating LTBI from aTB were distinct for HIV-uninfected and HIV-infected individuals (12 vs 54%, respectively) (Figure 5A). Our data were comparable to Adekambi et al.’s (17), despite the disparity in the cutoff value for these markers, which could be explained by flow cytometry technical differences. CD27 expression on Mtb-specific IFNγ+ cells also distinguished between LTBI and aTB irrespective of HIV infection (*p* = 0.0003, AUC = 0.85, 95% CI: 0.73–0.98, specificity: 77%, sensitivity: 79% for HIV− and *p* < 0.0001, AUC = 0.93, 95% CI: 0.84–1.02, specificity: 89%, sensitivity: 87% for HIV+) with comparable optimum cutoff values for HIV-uninfected and HIV-infected individuals (50 vs 43%, respectively, Figure 5B). Portevin et al. reported that CD27 expression defined as a ratio (CD27 MFI on total CD4+ cells/CD27 MFI on ESAT-6/CFP-10-specific IFNγ+ CD4+ cells) could be used to diagnose TB in children (13). We, thus, compared the ability of CD27 expressed as a percentage or as a ratio to distinguish latent from aTB in our study cohort. Figure S5 in Supplementary Material shows that both approaches had comparable sensitivity and specificity. Moreover, analysis combining HLA-DR and CD27 expression on Mtb-specific IFNγ+ CD4+ T cells did not improve the sensitivity or the specificity compared to HLA-DR alone (Figure S6 in Supplementary Material). Finally, we assessed the use of TNFα expression levels to delineate aTB from LTBI (Figure 5C). While the TNFα MFI on ESAT-6/CFP-10-specific IFNγ+ CD4+ T cells could distinguish aTB cases in both HIV-uninfected and HIV-infected individuals (*p* = 0.002, AUC = 0.79, and *p* = 0.007, AUC = 0.77, respectively); this measurement was less robust than HLA-DR or CD27. All the markers described above exhibited comparable ability to differentiate LTBI from aTB when measured on Mtb lysate-specific IFNγ+ CD4+ T cells (Figure S7 in Supplementary Material).

## Discussion

Assays based on host immune responses represent potential tools for TB diagnosis. However, their relative performance in HIV-1-infected persons has not been comprehensively assessed. Active TB has been inconsistently associated with specific cytokine production profiles, such as monofunctional TNFα+ CD4+ T cells (6), dual functional IFNγ+TNFα+ cells [(8–10) and this report], or cells co-expressing IFNγ, TNFα, and IL-2 (11, 18). By contrast, the measurement of the memory maturation profiles or the activation status of Mtb-specific CD4+ T cells has shown more consistent results (10, 13–18). Our study represents the first assessment of the relative performance of these markers, and their efficacy in HIV-infected individuals in a high burden TB and HIV co-epidemic.

In this study, HLA-DR expression on Mtb-specific IFNγ+ CD4+ T cells was the most robust marker to discriminate latent from aTB irrespective of HIV status. However, cutoff values for this marker differed significantly between HIV-uninfected and HIV-infected persons (12 vs 54%, respectively). On the other hand, CD27 expression (expressed as a percentage or MFI ratio) also correctly classified TB disease activity with good sensitivity and specificity but had the additional benefit of exhibiting comparable cutoff values for HIV-uninfected and HIV-infected individuals. This may be of importance when considering a diagnostic to be applied in countries with high HIV prevalence where many persons are frequently unaware of their HIV status. Interestingly, stimulation with Mtb lysate demonstrated the same sensitivity and specificity as ESAT-6/CFP-10 to distinguish aTB and LTBI. Even if such Mtb antigen formulation is potentially cross reactive to BCG or other mycobacteria, it restimulated greater Mtb responses (~4-fold) compared to Mtb peptides and may offer an advantage to detect aTB in individuals with low or no responses to ESAT-6/CFP-10.

In addition, we showed that in aTB cases, Mtb-specific CD4+ T cells had an elevated inflammatory potential (as measured by the amount of TNFα produced per cell). Nevertheless, this measurement performed poorly compared to phenotypic markers, further emphasizing that assessing the functional attributes of Mtb-specific CD4+ T cells may not yield useful TB biomarkers. However, one report showed that while the functional profile of ESAT/CFP-10-specific T cells (measured using a IL-2 ELISPOT assay) did not discriminate latent from aTB in children, elevated magnitude of IL-2 producing T cells targeting a different Mtb antigen (namely AlaDH protein) was strongly associated with aTB (22). This suggests that depending on their specificity, the functional capacity of Mtb-specific T cells are regulated differently in the context of active bacterial replication.

In terms of TB pathogenesis, an exacerbated inflammatory response could promote immune-mediated tissue injury and, thus, may contribute to pathology. Indeed, it was recently shown that increasing the per cell production of IFNγ in CD4+ T cells led to premature death in mice (23). It is of interest to note that cell functional profiles associated with their phenotypic characteristics, irrespective of pathogen specificity. This is consistent with published studies where cell memory maturation was related to a decrease in IL-2 production (24, 25) and KLRG1 expression linked to increased TNFα production (26, 27). This confirms that the degree of memory differentiation intrinsically regulates cell functional potential.

HIV is the best-recognized risk factor for TB disease. It is, thus, important to better understand the impact of HIV on Mtb immunity in order to define immunological features associated with increased TB risk. The most obvious immune defect caused by HIV is a progressive reduction in absolute CD4 cell numbers that correlates with increasing risk of TB (28). However, increased TB risk is observed even when reduction in CD4 counts is modest (early in HIV infection or in ART-treated patients) (28, 29). This suggests that HIV also affects Mtb immunity in a qualitative manner. Indeed, Mtb-specific CD4+ T cells are reported to be more permissive to HIV infection (30, 31), and we recently demonstrated that HIV alters the T helper differentiation potential of Mtb-specific CD4+ T cells (32). Less is known about the impact of HIV on the phenotype of Mtb-specific CD4+ T cells and conflicting data have been reported on the effect of HIV on the polyfunctional capacities of these cells (33–35). In this report, we did not observe major alterations in the differentiation, homing, or functional potential of Mtb-specific CD4+ T cells in HIV-infected individuals with LTBI compared to uninfected persons. This could be due to the fact that the LTBI/HIV+ group exhibited relatively well-preserved CD4 counts and suggests that HIV-induced dysfunction of Mtb-specific cells may only occur at a later stage of infection when HIV pathogenesis is more advanced. Conversely, HIV promotes the memory maturation of CMV-specific CD4+ T cells that associated with a reduction in IL-2 secretion capacity. These results suggest that HIV infection may impact memory CD4+ T cells differently based on their specificity.

In conclusion, our data show that CD27 and HLA-DR are promising biomarkers to discriminate latent from aTB, irrespective of HIV status. As T cell activation has been described as an immune correlate of risk for TB development in BCG-vaccinated infants (36), it will be of interest to further define whether HLA-DR expression on Mtb-specific CD4+ T cells could also be relevant to identify sub-clinical TB. Overall, our results aid in the development of blood-based assays for TB diagnosis, identified as a high-priority by the World Health Organization (37). As flow cytometry is finding increasing use in routine clinical tests, it is possible that a scalable whole blood-based assay assessing these markers could be developed.

## Ethics Statement

The study was approved by the University of Cape Town Human Research Ethics Committee (HREC No. 896/2014). All participants provided written informed consent.

## Author Contributions

Conceived, designed, and performed the experiments, and analyzed the data: CR. Contributed reagents, materials, and analysis tools: NB, RG, and RW. Wrote the paper: CR, WB, and RW. All authors approved the final manuscript.

## Conflict of Interest Statement

The authors declare that the research was conducted in the absence of any commercial or financial relationships that could be construed as a potential conflict of interest.
