# Supplementary figures and images for: Analysis of the Phenotype of Mycobacterium tuberculosis-Specific CD4+ T Cells to Discriminate Latent from Active Tuberculosis in HIV-Uninfected and HIV-Infected Individuals
Source: Front Immunol. 2017 Aug 10;8:968. doi: 10.3389/fimmu.2017.00968 (PMC5554366; doi:10.3389/fimmu.2017.00968)

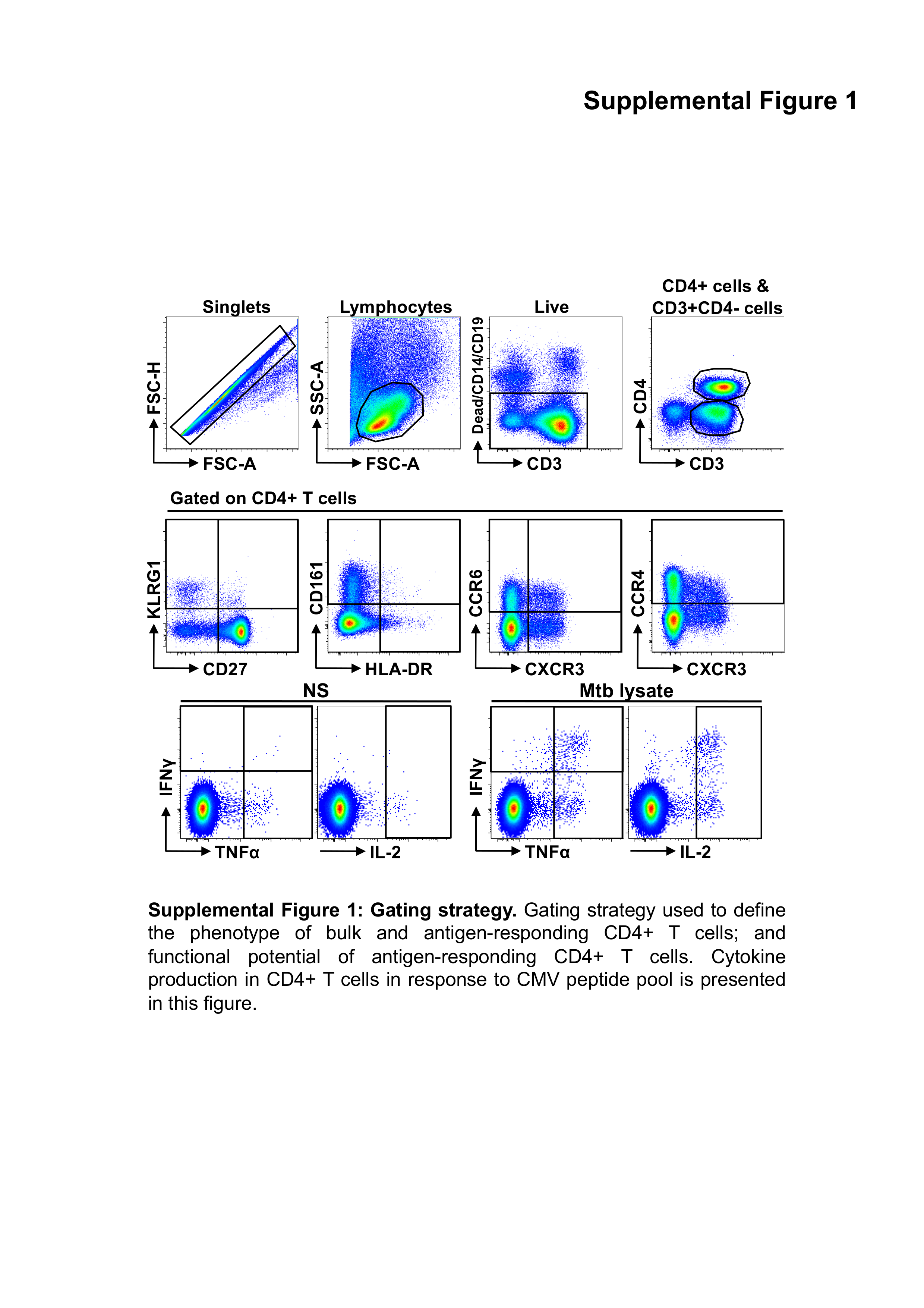

Supplement: Supplementary file 1 [file Image_1.TIF]

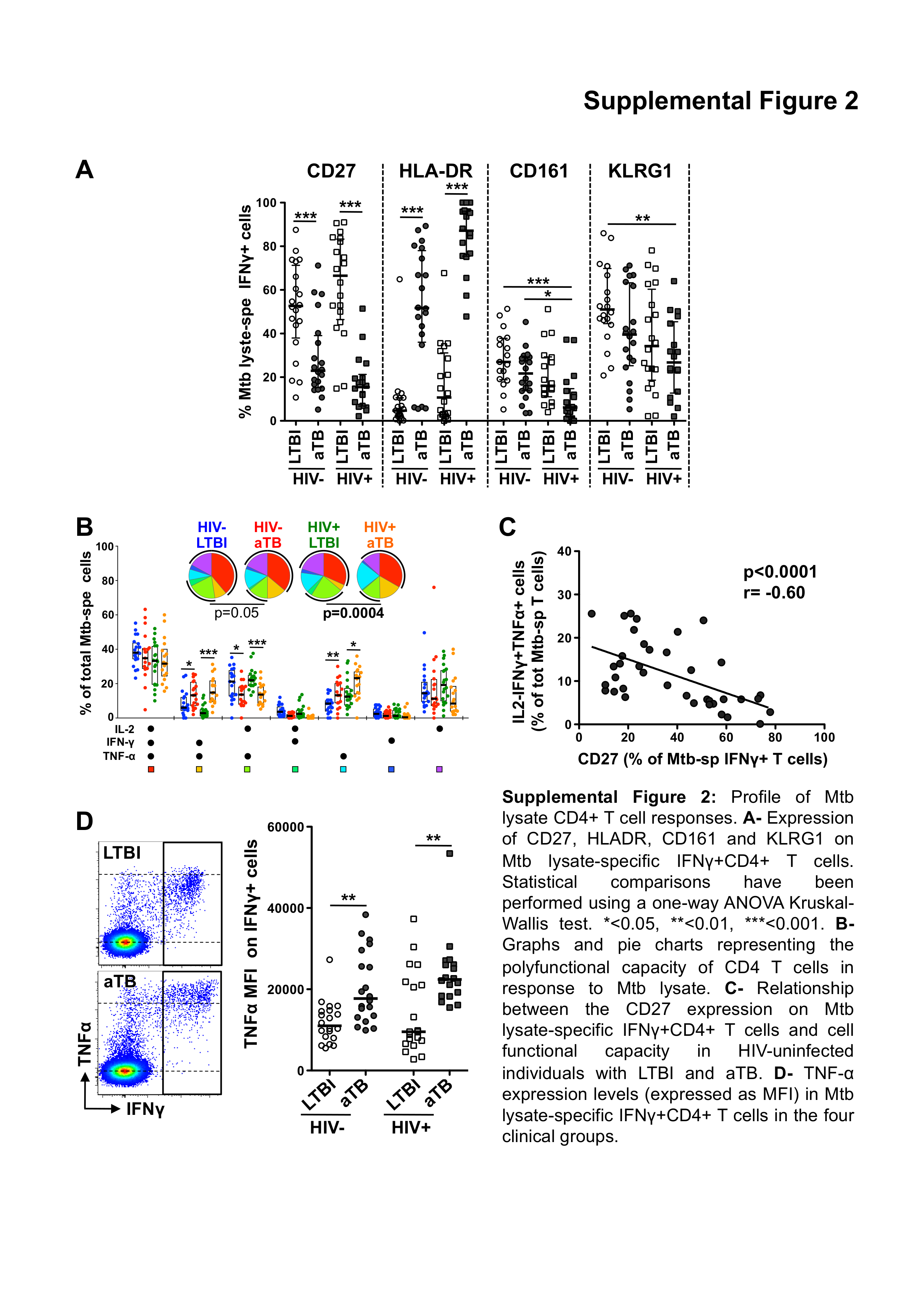

Supplement: Supplementary file 2 [file Image_2.TIF]

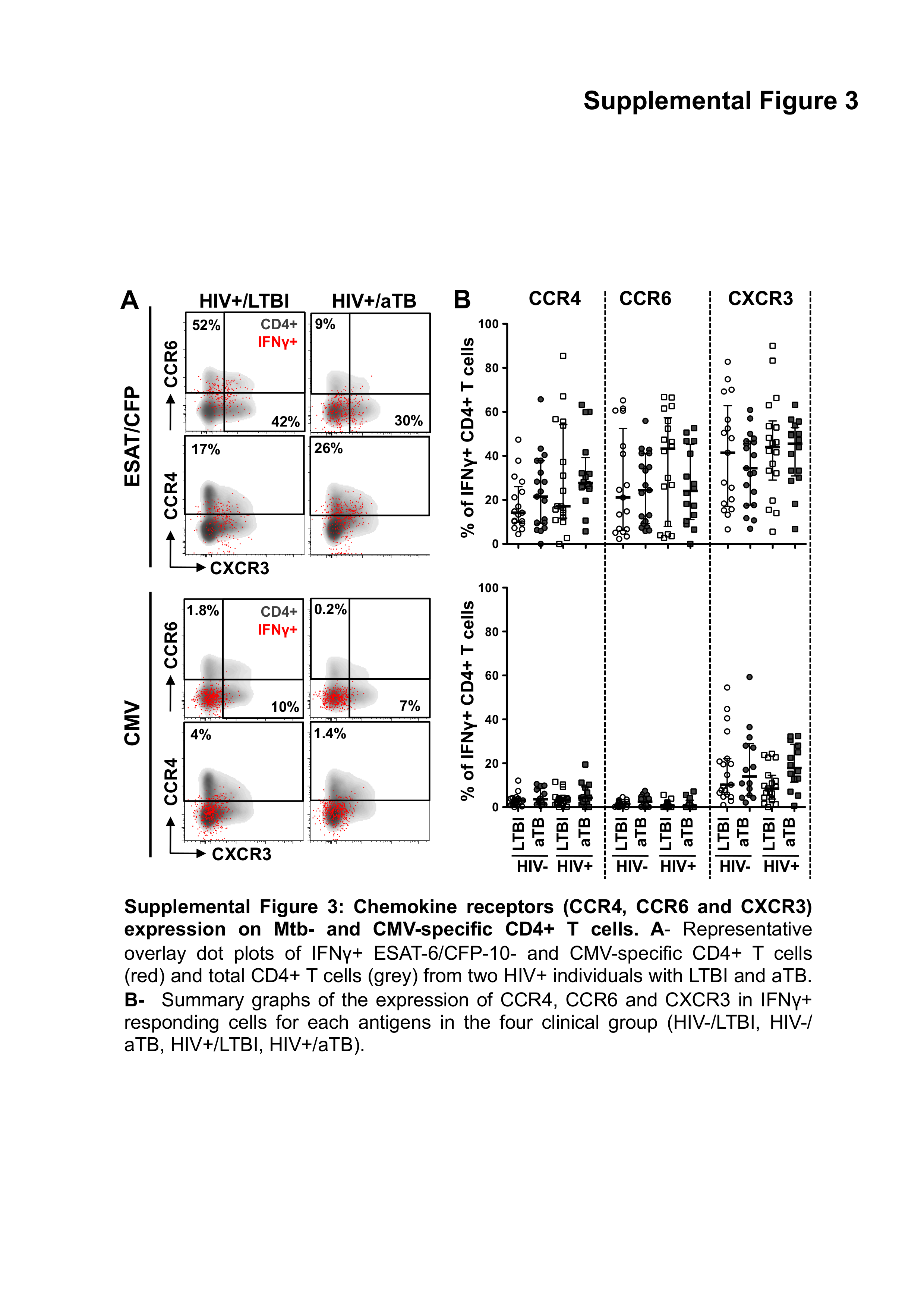

Supplement: Supplementary file 3 [file Image_3.TIF]

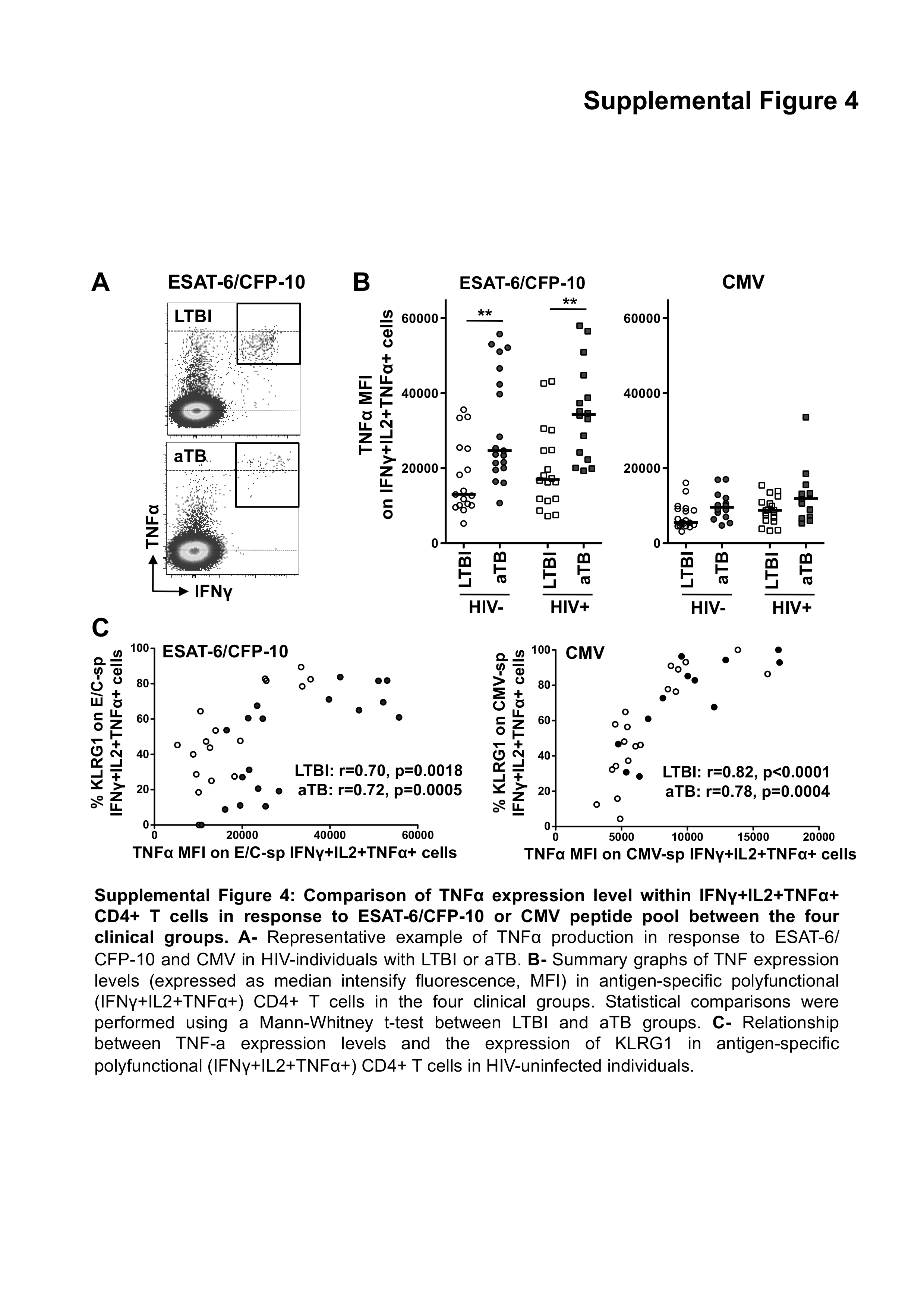

Supplement: Supplementary file 4 [file Image_4.TIF]

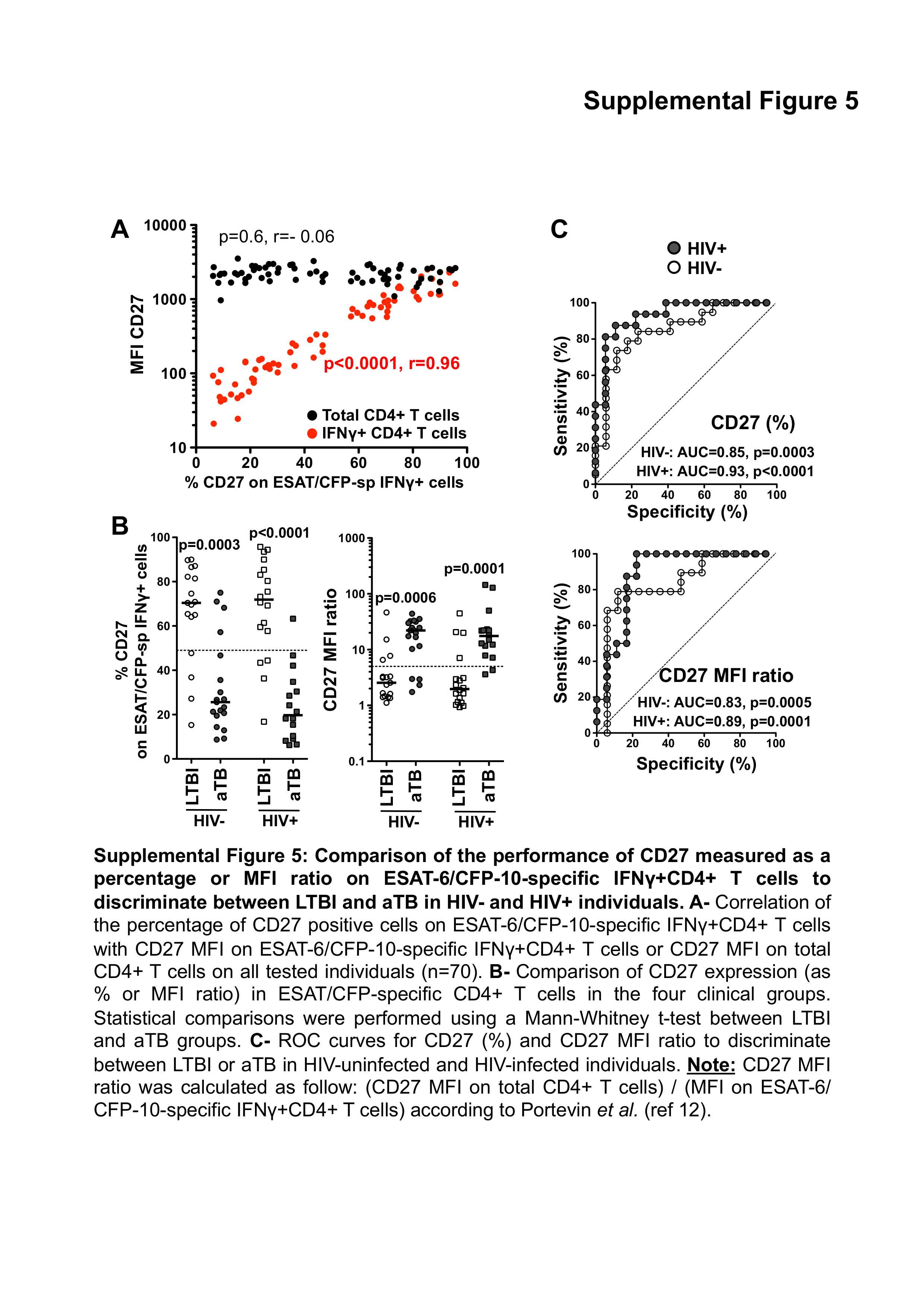

Supplement: Supplementary file 5 [file Image_5.TIF]

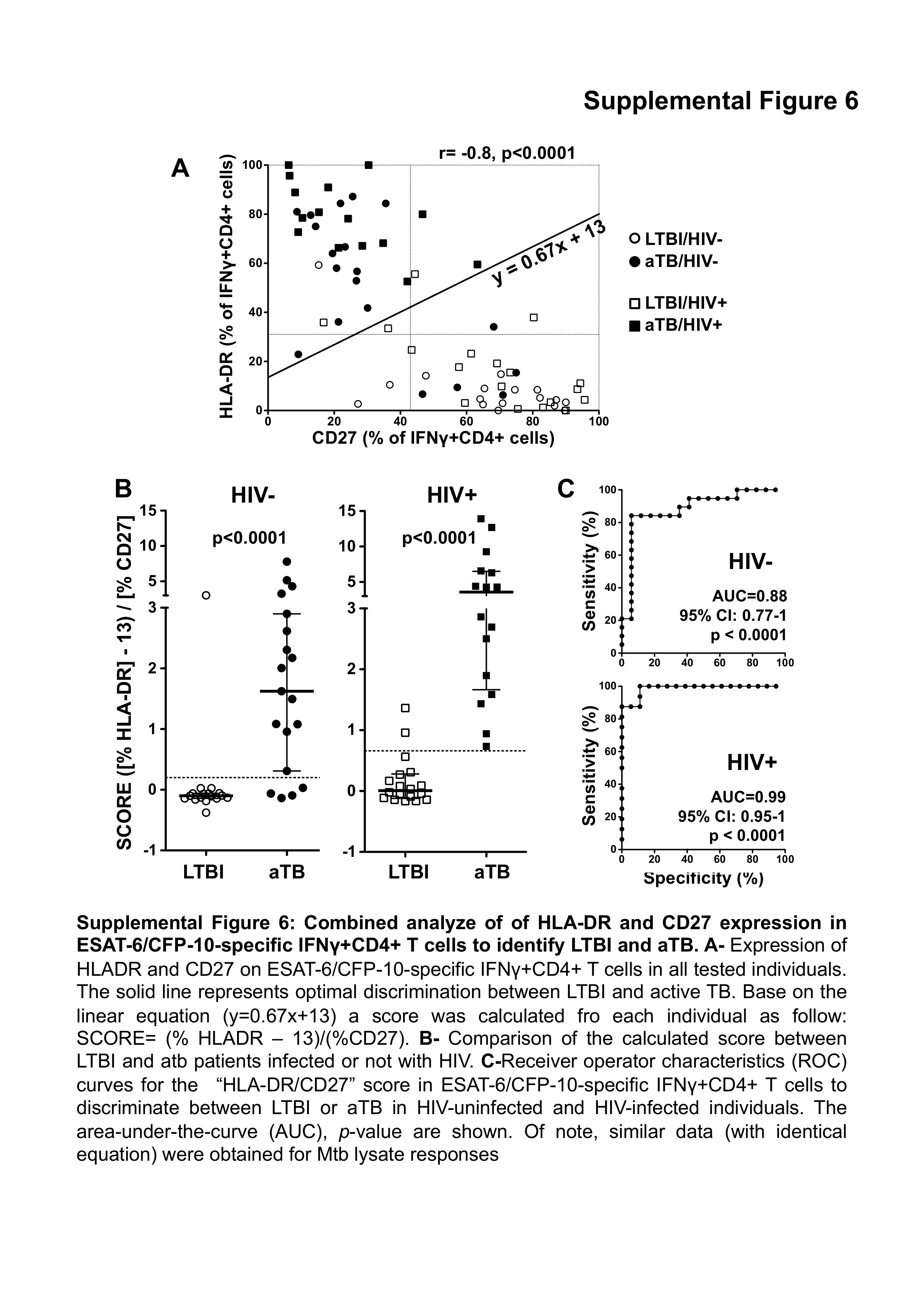

Supplement: Supplementary file 6 [file Image_6.TIF]

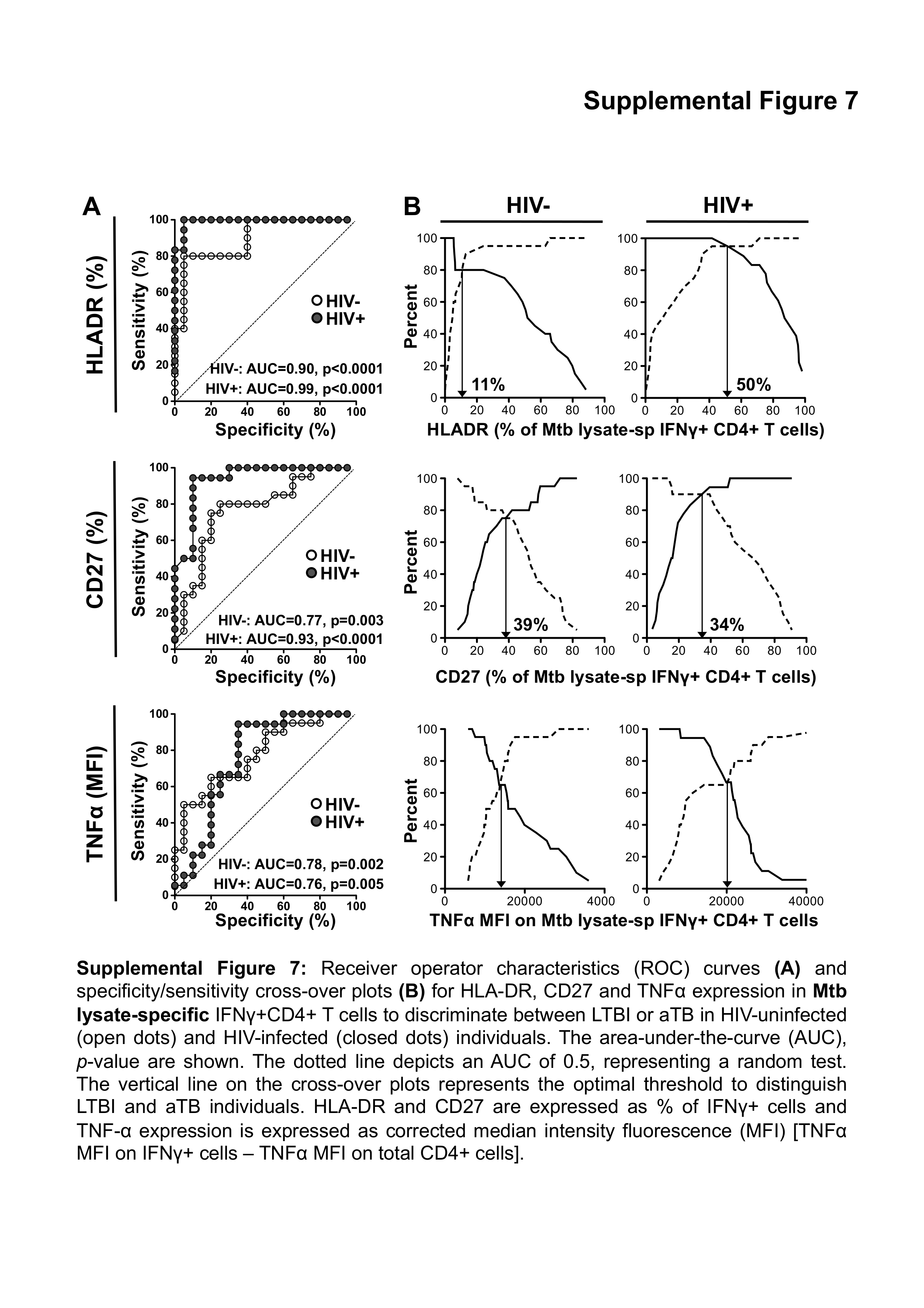

Supplement: Supplementary file 7 [file Image_7.TIF]
